# Microbiota and its antibiotic resistance profile in avocado Guatemalan fruits (*Persea nubigena* var. *guatemalensis*) sold at retail markets of Ibarra city, northern Ecuador

**DOI:** 10.3389/fmicb.2023.1228079

**Published:** 2023-09-07

**Authors:** Evelyn Angamarca, Pablo Castillejo, Gabriela N. Tenea

**Affiliations:** ^1^Biofood and Nutraceutics Research and Development Group, Faculty of Engineering in Agricultural and Environmental Sciences, Universidad Técnica del Norte, Ibarra, Ecuador; ^2^Grupo de Investigación en Biodiversidad, Medio Ambiente y Salud, Universidad de Las Américas, Quito, Ecuador

**Keywords:** avocado microbiota, metagenomics, multiple antibiotic resistance, pathogens, *Staphylococcus* spp.

## Abstract

**Introduction:**

Avocados are typically sold in unsanitary conditions at the retail markets in Ecuador, which can raise the risk of microbial contamination. These microorganisms could exhibit multi-antibiotic resistance (MAR), being a serious threat concern to human health. In this study, we aimed to evaluate the microbiota and its antibiotic resistance profile in avocado Guatemalan fruits (*Persea nubigena* var. *guatemalensis*), at ripe stage: immature, firm light green (ready to eat in 4 days), peel (AFPE) and pulp (AFPU), and mature intense green (ready to eat) peel (AMPE) and pulp (AMPU), to gain baseline information on the prevalence of MAR bacteria.

**Methods:**

Culture-independent (16S rRNA metagenomics) and culture-dependent approach (to detect specific indicator microorganisms) were used. Moreover, antibiotic susceptibility of selected target indicator bacteria was assessed providing information about the antibiotic resistance (AR) among the groups.

**Results:**

Based on 16S rRNA gene metagenomic analysis, over 99.78% of reads were classified as bacteria in all samples. Shannon diversity index varies from 1.22 to 2.22, with the highest bacterial population assigned to AFPE samples (1327 species). The highest microbial counts of indicator *Staphylococcus* spp. (STAPHY), *Enterobacter* spp. (ENT), and Listeria spp. (LIST), were detected in AMPE samples. Thirty percent of the selected STAPHYs, and 20.91% of *Enterobacter* (ENT) clones were resistant to various classes of antibiotics. The MAR index varies between 0.25 to 0.88 and was clone-, and fruit ripe stage-dependent.

**Conclusions:**

The results indicated that ready to eat avocados contained detectable levels of MAR bacteria, including methicillin resistant (MR)-STAPHY, which may act as a potential vector for the spread of antibiotic resistance. To achieve the increase of the production and marketing of Fuerte cultivar in Ecuador, it is vitally important to consider valuable strategies to protect the fruits at the early ripe stage in future. Thus, it is crucial to set up efficient control measures and develop coordinated strategies to guarantee the microbiological quality of the food.

## Introduction

Avocado (*Persea nubigena* var. *guatemalensis*), known as “Fuerte” among other high-demand crops such as bananas, mangos, and tree tomatoes or tamarillo (*Solanum betaceum*), is a part of the traditional food in the Ecuadorian diet and generally is consumed as fresh fruit (MAGAP, 2021). The main avocado-producing areas are Carchi, Imbabura, Pichincha, Tungurahua, Azuay, and Loja provinces (INIAP, 2014). It is a highly desired fruit in the world market due to its consistency, flavor, and nutritional value, in addition to its different uses in agro-industrial and pharmaceutical processes (Álvarez et al., 2015). In Ecuador, unlike other countries, avocados are produced all year round, with two harvest peaks fully defined from February to March and from August to September (INIAP, 2014). Fuerte variety is 99% for national consumption, while Hass is mainly exported. Fuerte variety is green in color both at harvest and at the time of consumption, with a pear-like shape and an opaque tone at its maturity. This variety production is hampered by several factors, including poor rootstalk, diseases, pests, abiotic factors, poor harvesting technology, improper handling, and postharvest diseases (Al-Kharousi et al., 2016). In addition, anthracnose disease caused by fungi is a major concern both in the field and postharvest (Kimaru et al., 2020). The fruit ripening stage is an important postharvest criterion in product selection as the state of maturity greatly influences the shelf life or storage ability (Sotomayor et al., 2016). Temperature is the most important factor in the reduction of the fruit ripening process as it influences the kinetics of metabolic reactions and deterioration, and the speed of ripening and ethylene production increased at high temperatures; thus, these fruits must be stored in low temperatures (12–14°C). Nonetheless, very low temperatures (below 0°C) can cause cold damage to the fruits (Castellanos et al., 2017).

The avocado Guatemalan fruits can be contaminated with pathogens during growth, harvest, transport, and handling (FAO, 2004). Compared with Hass, this cultivar is very perishable, and the fruits are harvested at the immature firm ripeness stage and then stored at room temperature. The fruit outer skin is no thicker than that of an apple and sometimes is woody in texture and low-frost resistant, therefore poor manipulation and storage conditions can increase the chances of microbial contamination with pathogenic bacteria at the postharvest stage. Although avocado microbiota is dominated by spoilage bacteria, yeasts, and molds (Bill et al., 2022), previous studies on the Hass variety indicated the presence of several pathogens such as *Pseudomonas aeruginosa, Staphylococcus aureus, Salmonella* spp., *Campylobacter* spp., *E. coli, Klebsiella* spp., and *Shigella* spp. (Gultie and Sahile, 2013; García-Frutos et al., 2020; Aliero et al., 2022). Recent literature review indicated that the presence of *S. aureus* in fruits and vegetables was indicative of poor personal hygiene and storage in contaminated settings (Balali et al., 2020). In addition, fruits and vegetables serve as a reservoir of microorganisms that harbor antibiotic resistance genes (Li et al., 2020). However, for developing management decision frameworks to reduce AR, in-depth studies on the diversity and prevalence of bacterial communities in fruits are crucial (Rahman et al., 2022). Traditional studies on fruit microbiota have focused on culturable bacterial groups, but these methods are limited as unculturable microorganisms cannot be identified (Cao et al., 2017). Thus, next-generation sequencing (NGS) technologies allow for studying this hidden microbial diversity in terms of different environmental parameters (Saminathan et al., 2018).

In Ecuador, Guatemalan avocado fruits are consumed as raw without further processing in different traditional dishes. At the retail open-air markets, poor storage conditions and inappropriate handling could lead to an increase in pathogenic bacteria contamination. In this study, we aimed to analyze the microbiota of ripe Guatemalan avocado fruits purchased from a local retail market in immature (firm) and mature (ready to eat) stages in both peel and pulp using the 16S rRNA-based metagenomic analysis. Moreover, a complementary conventional bacteriological analysis was performed to detect and count some target indicator pathogens (STAPHY, LIST, and ENT), total aerobe (AEROBE), and total yeasts/molds (YM). In addition, the presence of *E. coli* (EC) and *Shigella/Salmonella* (SHIGA/SALM) was evaluated. Furthermore, antibiotic susceptibility of selected target indicator bacteria was assessed providing information about the AR among the groups. STAPHY and ENT clones with the MAR index above 0.5 were sequenced to identify the species. This study provides a comprehensive overview of the bacterial prevalence and their antibiotic resistance profile in avocado Guatemalan fruits sold at the retail markets; further safety measures should be taken to control the contamination.

## Materials and methods

### Sample collection and processing

**Figure 1:**
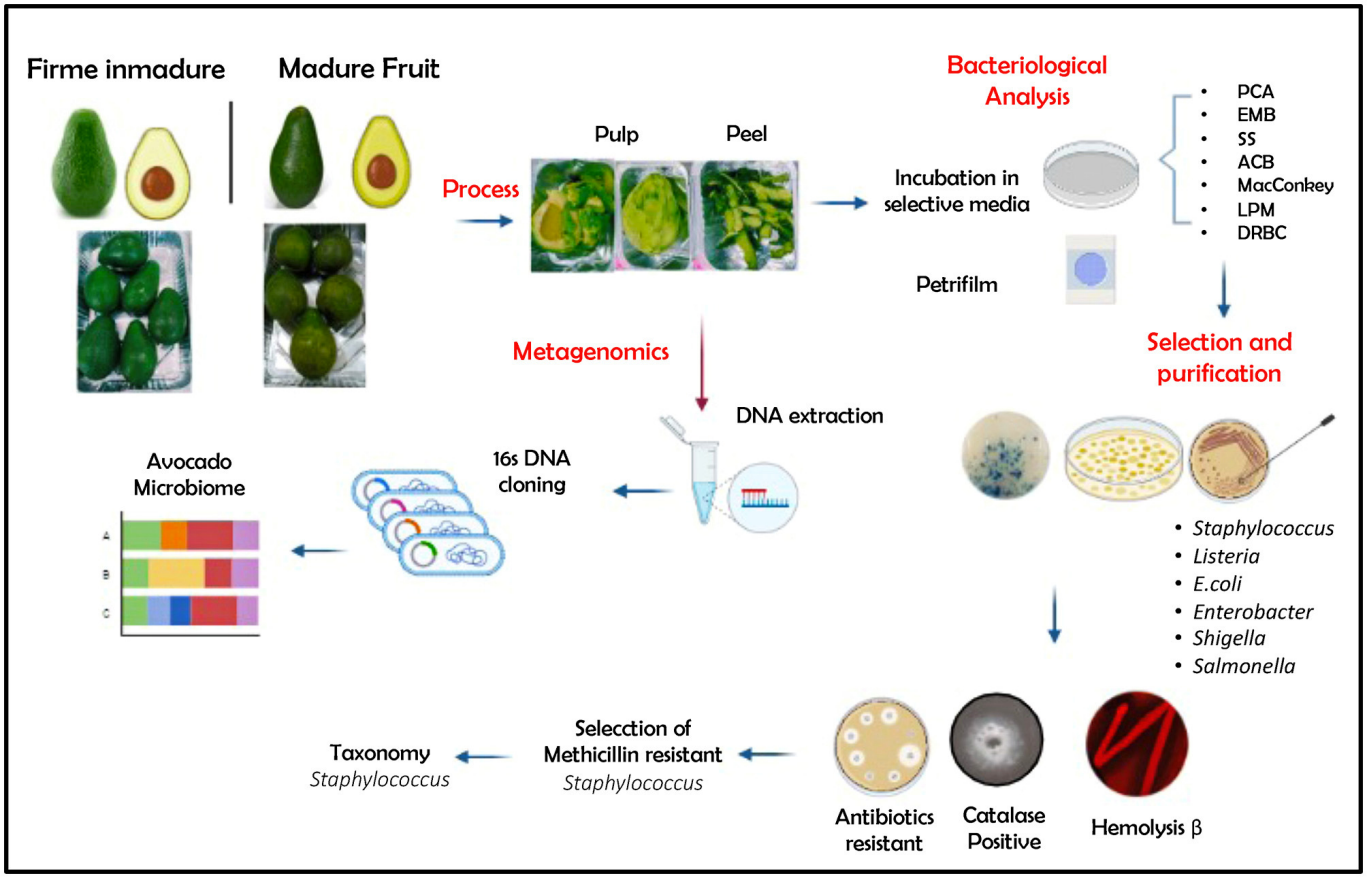
Overview of the workflow used in the analysis process. PCA, plate count agar; EMB, Eosin Methylene Blue Agar; SS, Salmonella–Shigella Agar; ACB, Aureus ChromoSelect Agar Base; MacConkey, Agar MacConkey; LPM, LPM Agar Base; DRBC, Dichloran Rose Bengal Chloramphenicol. Created with BioRender.com.

Fruits of Guatemalan avocado were purchased from a retail market of Ibarra city (capital of Imbabura province, northern Ecuador) in two ripening stages: an immature firm (AF) recognized as light green fruit (ready to eat in 4 days) and mature intense green (AM) with soft skin fruit (ready to eat). The fruits with no visible damage (5 fruits × 2 stages × 3 repetitions: total 30 fruits) were taken and transported to the laboratory for further analyses. The fruits were washed twice with tap and distillate water before further manipulation. In addition, a bulk of peel and pulp (CPE) from visibly spoiled fruits (5 fruits × 3 repetitions: total 15 fruits) were used for comparison. The cutting utensils (knife, spatula) and board were surface-sterilized with 70% ethanol to avoid cross-contamination. Figure 1 shows an overview of the workflow used in the analysis process.

### Culture-independent assay: 16S rRNA gene metagenomics

#### DNA extraction

Exocarp (peel) from a pool of 15 fruits per stage (AFPE: peel immature firm light green fruit and AMPE: peel from mature intense green (ready to eat), ~200 g) was collected and ground gently with liquid nitrogen prior to DNA extraction. Before DNA extraction, the remaining endocarp samples (pulp) (> 500 g) from immature firm light green fruit (AFPU) and mature intense green (AMPU) were placed in a sterile glass dish, cut into small pieces, and mixed. Genomic DNA was isolated using a power food microbial DNA isolation kit, following the instructions of the manufacturer (MO BIO Laboratories, USA). DNA quality and quantity were determined using a spectrophotometer (Nanodrop 1100, Colibri, Berthold Technology UK Ltd, UK). Similarly, DNA was extracted from CPE samples. Isolated DNA was stored at −20°C and used in further analysis.

#### Library construction and sequencing

Metagenomic sequencing was performed on an Illumina NovaSeq platform (paired-end 150 bp reads) by Biosequence (custom design assay, Quito, EC), following a comprehensive workflow that combines a benchtop sequencing system, on-board primary analysis, and secondary analysis using MiSeq Reporter or BaseSpace (Illumina, USA). The 16S rRNA V3-V4 region was amplified with the bacterial primers 341F (5'-CCTACGGNGGCWGCAG-3') and 805R (5'-GACTACHVGGGTATCTAATCC-3') (Klindworth et al., 2013) and added Illumina sequencing adapters and dual-index barcodes to the amplicon target. All polymerase chain reactions (PCRs)involved the KAPA HiFi HotStart ReadyMix (Sigma–Aldrich, USA). A washing step using magnetic beads was applied to purify the 16S, V3, and V4 amplicons from free primers and primer dimer species. Using the full complement of Nextera XT indices, up to 96 libraries were pooled for sequencing. The sequence of MiSeq using paired 300-bp reads and MiSeq v3 reagents, and the ends of each read are overlapped to generate high-quality, full-length reads of the V3 and V4 regions. Library preparation consisted of adding indices to each end of the previously obtained amplicons. Indices are unique sequences that are assigned to all amplicons in the same sample to distinguish them from amplicons in other samples, allowing multiple samples to be sequenced in parallel and independent data for each (Illumina workflow). The second wash was performed using magnetic beads to clean the final library. Finally, the purified libraries were quantified and qualified to determine their suitability for sequencing.

#### Diversity analysis of 16S amplicons

The metagenomics workflow is a secondary analysis option built based on the MiSeq Reporter (on-system software) or available on BaseSpace (cloud-based software) (Illumina, USA). FASTq files were subjected to a quality and filtering process to guarantee taxonomic classification. For taxonomic classification, an implementation of a high-performance algorithm of the Ribosomal Database Project (RDP) (https://benjjneb.github.io/dada2/training.html) classifier was used (Wang et al., 2007). For taxonomy assignment, 16S rRNA gene sequences with DADA2 format for bacteria and archaea were used (version 4.2) (Alishum, 2021). The follow-up study involved the use of UPARSE, and sequences with 97% similarity were assigned to the same operational taxonomic units (OTUs) (Edgar, 2013). The phylogenetic interaction of different OTUs, the differences in the middle of the dominant species in the samples (groups), and the alignment of diverse sequences were analyzed on the core set dataset using PyNAST v1.2 (Caporaso et al., 2010). The Shannon diversity index was used to estimate the diversity of species within the groups (Krebs, 2014). Heatmaps and hierarchical clustering with the unweighted pair group method (UPGMA with Euclidean distance) were performed to evaluate the change in community composition at the phylum and genus levels. Venn diagrams were created to examine the intersection of the bacterial families between peel (AFPE vs. AMPE), pulp (AFPU vs. AMPU), and CPE (visibly spoiled) fruits. These analyses were performed on the bioinformatics platform (https://www.bioinformatics.com.cn/en).

### Culture-dependent assays: targeting specific indicator microorganisms

The bacteriological analysis was performed as previously described (Tenea et al., 2023). In brief, the exocarp (AFPE and AMPE samples) was gently removed, chopped into small pieces, and mixed, and 25 g/sample was inoculated in pre-enrichment buffered peptone water (0.1%), homogenized, and incubated for 4 h at 37°C. Similarly, 25 g pulp/sample (AFPU, AMPU) was used. In addition, following the same procedure, CPE sample (visibly spoiled) was analyzed. After incubation, decimal dilutions made with sterile water were inoculated on 3M Petrifilm Aerobic (3M Science Applied to Life, Detroit, MI, USA), to determine the total aerobic microbial population (37°C, 48 h). To detect and differentiate the presumptive presence of SALM/SHIGA, aliquots (100 μl) were plated on Shigella–Salmonella (SS) agar (Difco, Detroit, MI, USA) and incubated for 48 h at 37–40°C. The presence of SALM was confirmed as previously described (ISO, 2002). Independent experiment aliquots (100 μl) were placed on Chromocult Coliform agar (Merck Millipore, Kenilworth, NJ, USA), to determine the total coliforms and EC, and eosin methylene blue (Difco, Detroit, MI, USA), to detect the presence of ENT and EC. In addition, 3M Petrifilm Yeast and Mold Count Plates (3M Science Applied to Life, Detroit, MI, USA) were used for the enumeration of YM (incubation at 25–28 °C for 7 days). The presence of STAPHY was determined in Brilliance Staph 24 agar medium (Oxoid Limited, Wade Road, Basingstoke, Hampshire, UK) (ISO, 2018), whereas the presence of LIST was evaluated on GranuCult FRASER broth (Merck, Millipore, Kenilworth, NJ, USA) (ISO, 2017). The experiments were run in triplicate, and the microbial counts were expressed as logCFU/g.

### Physicochemical analysis of fruits

The pH was determined using a pH meter (SevenCompact S210, Mettler Toledo LCC, Columbus, OH, USA). Total acidity, expressed as a percent of citric acid, was determined by titrating with 0.01 M NaOH to pH 8.2 as described (Tenea et al., 2023). The total soluble solids (°Brix) was evaluated using a digital refractometer to determine the total sugar in fruits.

### Antibiotic profile analysis of selected clones

A total of 52 random colonies from both peel and pulp (8–10 colonies/stage/indicator) were screened for antibiotic susceptibility according to the Kirby–Bauer disk diffusion procedure (CLSI, 2021) against the following panel of antibiotics: amoxicillin (AMX: 25 μg), ampicillin (AM: 10 μg), gentamicin (CN: 10 μg), kanamycin (K: 30 μg), tetracycline (TE: 30 μg), and cefuroxime (CXM: 30 μg). *E. coli* ATCC25922, *S. enterica* subsp. *enterica* ATCC51741, *L. monocytogenes* ATCC19115, *Enterobacter* spp. UTNEnt1 (a laboratory AR strain isolated from strawberry), *S. aureus* ATCC43300 (methicillin sensible), and *S. aureus* ATCC1026 (methicillin-resistant) strains were used as references. The microbiological breakpoints reported by the FEEDAP standards were used to categorize the clones as susceptible, intermediary, or resistant (EFSA, 2012). The Scan500 was used to determine the inhibitory halos automatically (Interscience, Fr). The percentage of resistance was determined as the number of total bacteria resistant/number of total isolates tested. The MAR index was calculated as the ratio between the number of antibiotics that an isolate is resistant to and the total number of antibiotics the organism is exposed to.

### Taxonomy assignment of STAPHY and ENT clones

The 16S rRNA gene sequencing was used for the taxonomical assignment of the selected MR clones (9) and ENT (2) clones with a MAR index above 0.50 (Macrogen Inc., Korea, custom service). The PCR was performed using 27F 5' (AGA GTT TGA TCM TGG CTC AG) 3' and 1492R 5' (TAC GGY TAC CTTGTT ACG ACT T) 3' primers (Weisburg et al., 1991). The PCR reaction was carried out with EF-Taq (SolGent, Korea), with the following protocol: activation of Taq polymerase at 95°C for 2 min, 35 cycles of 95°C for 1 min, at 55°C and 72°C for 1 min, and extension of 10 min at 72°C. The amplification products were purified on a multiscreen filter plate (Millipore Corp., Bedford, MA, USA). The sequencing reaction was performed using a PRISM BigDye Terminator v3.1 cycle sequencing kit. The DNA samples containing the extension products were added to Hi-Di formamide (Applied Biosystems, Foster City, CA). The sequencing was conducted using 785F 5' (GGA TTAGAT ACC CTG GTA) 3' and 907R 5' (CCG CAA TTC MTT TRA GTT T) 3' primers, which are the inter-primers of 16S RNA V3 region (Muyzer et al., 1993). The mixture was incubated at 95°C for 5 min, followed by 5 min on ice, and then analyzed by an ABI Prism 3730XL DNA analyzer (Applied Biosystems, Foster City, CA). A fast homology search of the sequences was conducted using the megablast algorithm against the 16S ribosomal RNA database at NCBI (http://www.ncbi.nlm.nih.gov/BLASTN), as implemented in Geneious Prime 2020.2.3 (Kearse et al., 2012). This first search was used to obtain a maximum of 100 hits and associated search quality parameters that provided an initial reference for taxonomic classification. A final taxonomic assignment was made by the RDP Bayesian classifier algorithm (Wang et al., 2007) with 100 bootstrap replicates and a K-mer of size 8, as implemented in the function “accurate, high-resolution sample inference from the amplicon sequencing data” (assigned taxonomy) of the DADA2 package (Callahan et al., 2020).

## Results and discussion

### Bacterial community distribution

**Figure 2:**
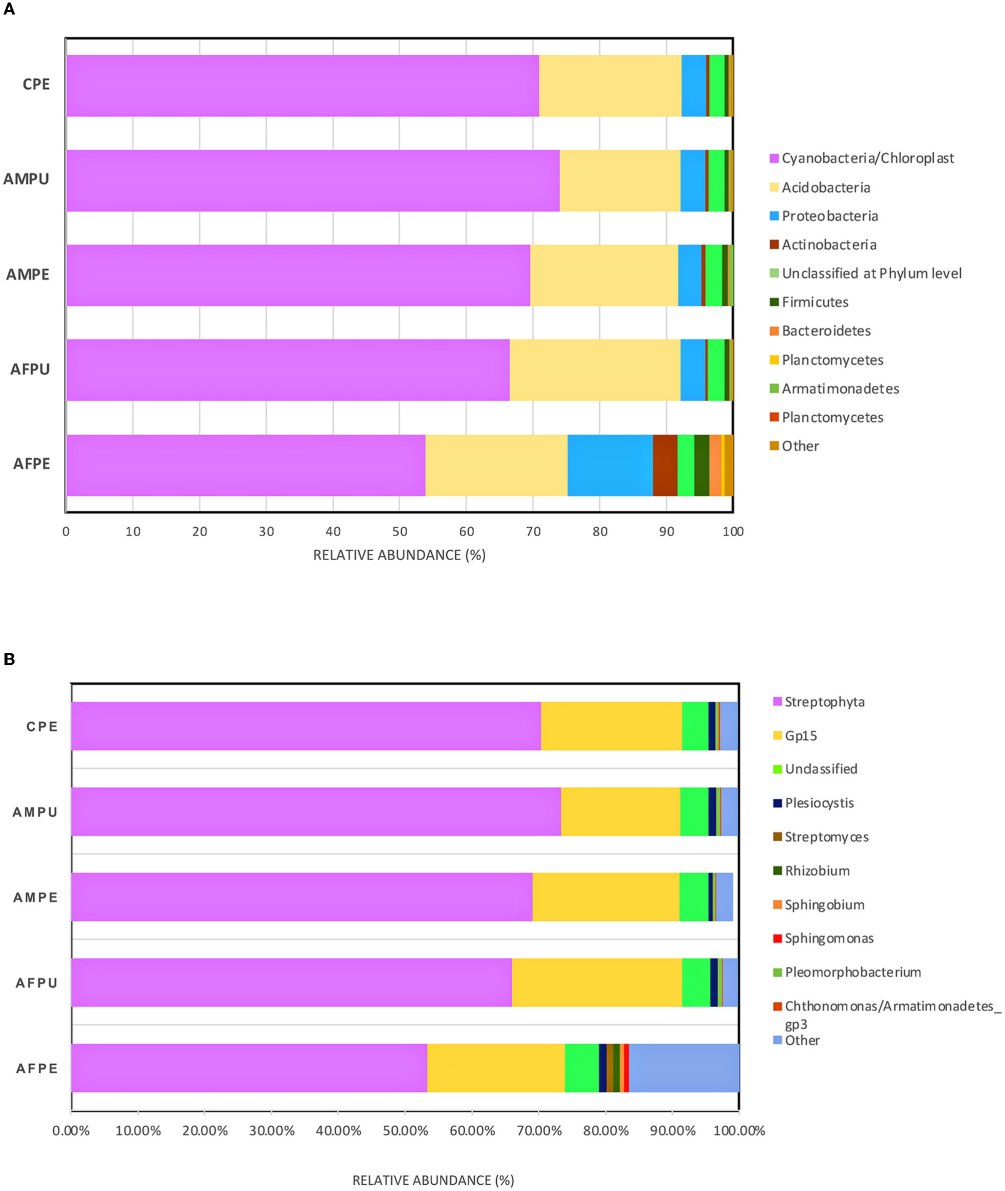
Distributions of bacterial community structures at different taxonomic levels. **(A)** The bar chart shows the relative abundance of bacterial community structures at the phylum level. **(B)** The bar chart displays the relative abundance of bacterial community structures at the genus level. AFPE, immature firm light green peel (ready to eat in 4 days); AFPU, pulp from immature firm light green fruits (ready to eat in 4 days); AMPE, peel from mature intense green (ready to eat) fruit; AMPU, pulp from mature intense green (ready to eat) fruit; CPE, bulk of peel and pulp from visibly spoiled fruits. The ”Other“ category in this pie chart is the sum of all classifications with <0.15 % abundance.

**Figure 3:**
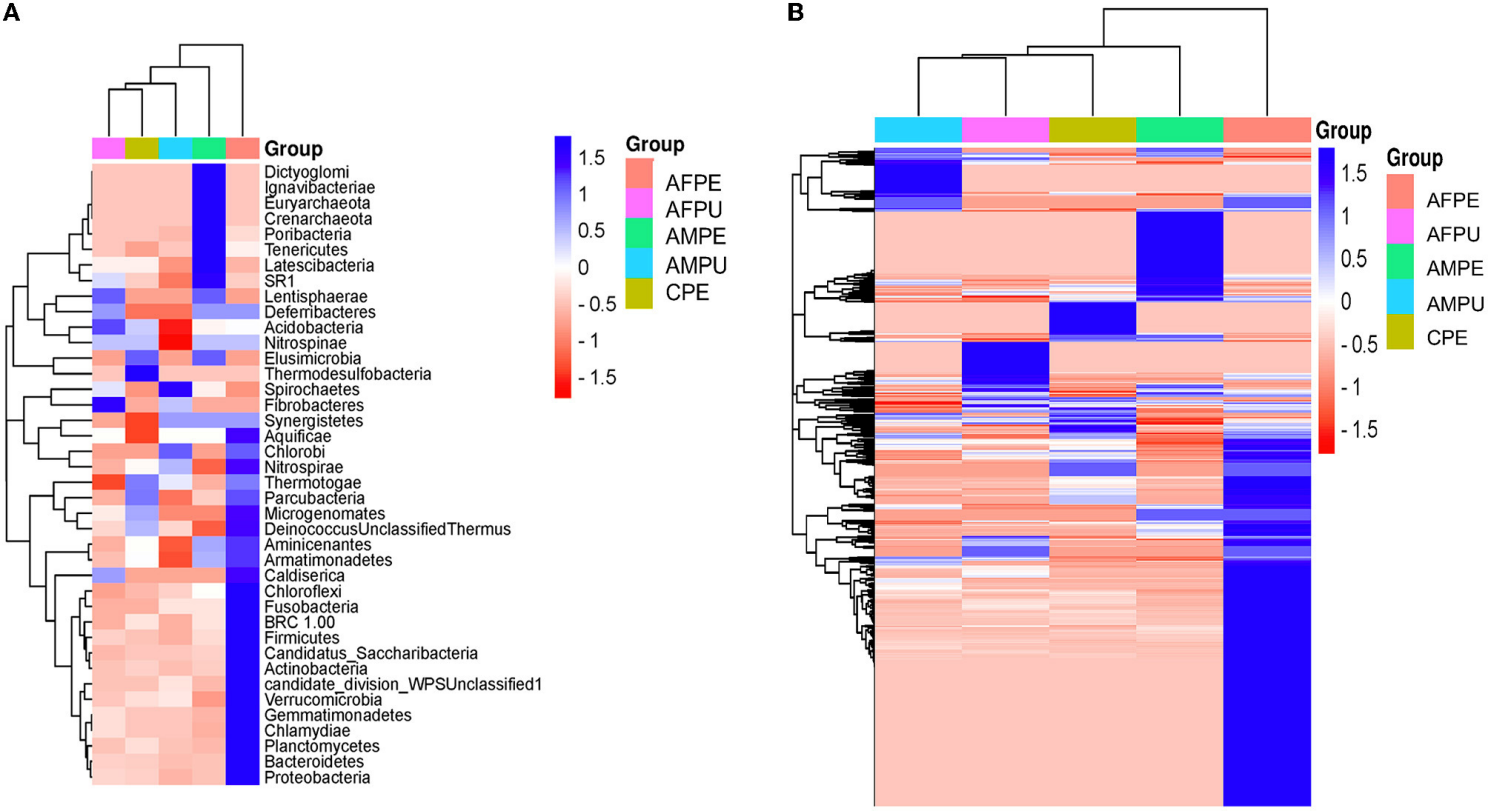
Heatmaps and hierarchical clustering of the most abundant phylum **(A)** and genus **(B)** levels. Color shading indicates the prevalence of each bacterium phylum and genus among samples (blue intense: most abundant; red intense: less abundant). AFPE, immature firm light green peel (ready to eat in 4 days); AFPU, pulp from immature firm light green fruits (ready to eat in 4 days); AMPE, peel from mature intense green (ready to eat) fruit; AMPU, pulp from mature intense green (ready to eat) fruit; CPE, bulk of peel and pulp from visibly spoiled fruits.

**Figure 4:**
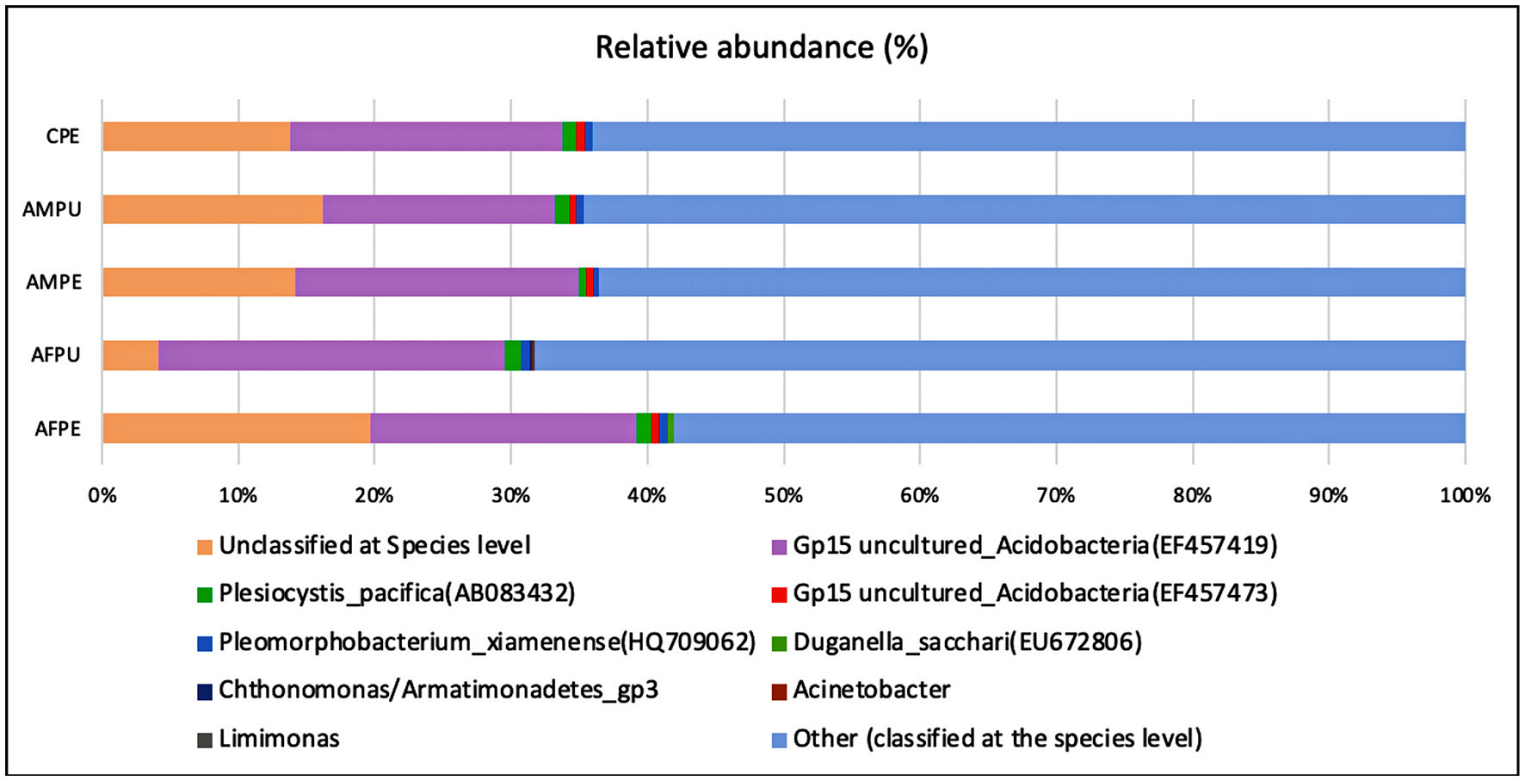
Distributions of bacterial community structures at the species level. This bar chart shows the relative abundance of the top 10 classification results within each taxonomic level. AFPE, immature firm light green peel (ready to eat in 4 days); AFPU, pulp from immature firm light green fruits (ready to eat in 4 days); AMPE, peel from mature intense green (ready to eat) fruit; AMPU, pulp from mature intense green (ready to eat) fruit; CPE, bulk of peel and pulp from visibly spoiled fruits. Category names appearing in parentheses are due to incomplete entries in the taxonomic database. They occur when a lower level category is specified, but the name for this higher level category is empty. The ”Other“ category in this pie chart is the sum of all classifications with >0.50 % abundance.

The results revealed ~56,228 and 58,111 clean reads for AFPE and AFPU, while 53,104 and 45,236 reads were found in AMPE and AMPU, respectively (Table 1). In total, 99.85% of reads for AFPE/AMPE and 99.94% for AFPU/AMPU samples were classified as bacteria. Alike, 60,296 reads (99.78%) were classified for CPE sample. At the phylum level, Cyanobacteria, Acidobacteria, and Proteobacteria were the most abundant among the groups. The total phylum-level taxonomic categories identified were 33 for AFPE and AFPU, 35 for AMPE, 28 for AMPU, and 29 for CPE. The top 10 abundant bacterial community structures at the phylum level are shown in Figure 2A and Supplementary Table 1. A major abundant class of “chloroplast bacterial genome” was observed in AMPU and CPE samples. The plastid genome contains a bacterial genome signature explaining the high abundance of the Cyanobacteria phylum (Robinson et al., 2022). In addition, Firmicutes were the most abundant in AFPE (2.26%), while Bacteroidetes were less abundant in the AMPU (0.15%) samples (Supplementary Table 1). Early metagenomic studies indicated that Proteobacteria, Acidobacteria, Bacteroidetes, and Firmicutes were the most abundant phyla in grapes (Zarraonaindia et al., 2015), while Firmicutes, Actinobacteria, and Proteobacteria were more abundant in melon pulp *Cucumis melo* L. (Glassner et al., 2018). In addition, the abundance of Proteobacteria in watermelon was linked to the ability of the fruit to utilize a wide variety of carbohydrates, amino acids, and lipids (Xia et al., 2015). The relative abundance and the top 10 categories at the genus level are shown in Figure 2B and Supplementary Table 2. A total of 770 and 404 reads were categorized for AFPE and AFPU samples, whereas 401 and 381 reads were categorized for AMPE and AMPU samples. A total of 422 reads were identified in CPE samples. To detect changes in bacterial community composition and visually compare the overall absolute abundance among the groups at the phylum and genus levels, heatmaps and hierarchical clustering were developed (Figures 3A, B). The AMPE and AMPU were grouped into the same clade with CPE samples, suggesting that the mature and firm fruits differ in bacterial composition. This may correlate with the differences in the physicochemical parameters with the high value of total solids detected in the mature stage (Supplementary Table 3). Most likely, this microenvironment allows bacteria and other germs to invade, adapt, and survive during storage. According to the findings, Gp15, a clade of unidentified reads, and *Streptophyta*, a clade of plants, were the most abundant categories. Recent studies investigating the hospital airborne microbiome indicated that Gp15, *Pseudomonas, Staphylococcus, Corynebacterium*, and *Acinetobacter* genera can be responsible for different types of nosocomial infections (Perrone et al., 2022). All sample groups contained pleomorphic bacteria, such as those generally associated with *Staphylococcus*; however, AMPU and AFPE samples showed 0.60% relative abundance of these species. *Streptomyces*, the most widespread and perhaps most important genus of Actinomycetes, was found in AFPE samples. The species of this genus are a good source of bioactive substances, antibiotics, and extracellular enzymes (Olanrewaju and Babalola, 2019). Although low abundant (0.80%), *E. coli* and *Shigella* were detected in AMPE samples (Supplementary Table 2). Additionally, using the Venn diagram, 20 (19.8%) and 18 (31%) families were shared between peel and pulp, respectively (Supplementary Figure 1). While Enterobacteriaceae were found in all samples (firm, mature, and spoilage fruits), bacteria belonging to the Marinilabiliaceae, Vibrionaceae, and Helicobacteraceae families were detected in AMPE. A larger sample size and additional research are required to confirm these findings and provide a biological context; hence, no precise role could be assigned. At the species level, the AFPE group showed the highest Shannon index diversity of 2.22 (1,327 species), while the lowest species diversity (1.21) (363 species) was detected within the AMPE group (Table 1). AFPE had the highest number of designated species, and at this point, no potential human pathogens had been found. The relative abundance of reads assigned at the species level is shown in Figure 4. *Staphylococcus strepanovicii* was prevalent in AMPE and CPE samples (relative abundance of 0.12%), whereas *S. gallinarum, S. warneri*, and *S. pasteuri* were prevalent in AMPE samples (relative abundance of 0.07, 0.13, and 0.12%, respectively) (Supplementary Figure 2). *S. strepanovicii* was mainly associated with mammals. *S. gallinarum* spp. are opportunistic human pathogens that have primarily been identified in poultry (Shi et al., 2015). *S. warneri* was detected as an endophyte in the peel of apples from major commercial markets in Tamil Nadu, India (Phukon et al., 2013). In addition, in less abundance (0.12%), *Weissella oryzae* was detected in the AFPE and AFPU samples, while *Lactobacillus fermentum* was detected in AFPE samples. Thus, the 16S metagenome study is the first to provide a diagnosis of bacterial diversity in ripe and unripe Guatemalan avocados; nonetheless, the composition of fungal communities associated with postharvest avocados is recommended. These results may contribute to a better understanding of the microbial composition of harmful bacteria, which will aid in the development of safety measures to prevent their spread before selling. The microbial 16S rRNA sequences were deposited in the Sequence Read Archive (SRA, https://www.ncbi.nlm.nih.gov/sra/) under accession code PRJNA972543 (15 May 2023).

**Table 1:** Percentage of reads classified to kingdom and genus levels, and the Shannon species diversity index.

**Sample ID**	**Number Reads passing quality filtering**	**% Reads classified to kingdom**	**% Reads classified to genus**	**Shannon species diversity**	**Number of species identified**
AFPE	56,228	99.85	94.81	2.22	1,327
AFPU	58,111	99.94	95.83	1.31	417
AMPE	53,104	99.85	95.64	1.21	363
AMPU	45,236	99.84	95.73	1.28	375
CPE	60,296	99.78	96.08	1.29	481

AFPE, immature firm light green peel (ready to eat in 4 days); AFPU, pulp from immature firm light green fruits (ready to eat in 4 days); AMPE, peel from mature intense green (ready to eat) fruit; AMPU, pulp from mature intense green (ready to eat) fruit; CPE, bulk of peel and pulp from visibly spoiled fruits.

### Indicator microorganisms' prevalence

In this study, bacteriological analysis revealed a high content of indicator bacteria in the mature ripe stage than in the immature (firm) stage (Table 2). The highest number of total aerobes was detected in AMPE (3.62 ± 0.18 logCFU/g), AMPU (3.68 ± 0.37 logCFU/g), and CPE (3.85 ± 0.38 logCFU/g) samples, suggesting that during shifting from immature to mature stage, these fruits become susceptible to contamination. Nonetheless, the increase in microbial population could be part of fruit natural flora. Interestingly, EC was detected in AMPU only, while SALM and SHIGA were detected in AMPE and AMPU samples. Early research indicated the presence of *Salmonella* and *Shigella* species in avocado mature fruits collected from the market of Northern Nigeria (Shiferaw and Kibret, 2018). In addition, Coetzee et al. (2017) reported the presence of *E. coli* and *Salmonella* spp. in avocado fruits collected from Southern Nigeria. Moreover, the highest amount of STAPHY and LIST was detected in mature (AMPE and AMPU) and spoiled (CPE) avocado fruits (Table 2). Recent research indicated a high frequency of occurrence (29.2%) of *S. aureus* in spoiled avocado fruits obtained from three market locations in Sokoto city, Nigeria (Aliero et al., 2022). In addition, avocado fruits purchased from retail markets in Guadalajara (Mexico) contained *E. coli, Salmonella* spp., *Listeria* spp., and *L. monocytogenes*, according to previous studies (Shiferaw and Kibret, 2018; García-Frutos et al., 2020). Avocados are regularly sold in bulk in retail markets in Ecuador, where they are exposed to potential sources of pathogens while being kept at outdoor temperature for several days. More likely, these pathogens may adhere, survive, and form biofilms under these circumstances. Although there is no proof of how these bacteria can reach the pulp, we suspect that during handling, germs may be transported to the pulp (comestible part) from the spoon used to remove the peel. While yeasts were not detected, high content of molds was found in the AMPE, AMPU, and CPE samples (Table 2). The differences in physicochemical properties may be responsible for the divergence in the prevalence of some microbe species in these fruits. Altogether, our analysis supported earlier research (García-Frutos et al., 2020; Aliero et al., 2022; Cabrera-Díaz et al., 2022), linking the fruit contamination with storage conditions, the use of microbiological hazardous containers, poor handling techniques, and unsanitary market conditions.

**Table 2:** Prevalence of microbial indicator counts in avocado fruits.

**Sample code**	**Total aerobes**	***E. coli* spp**.	***Enterobacter* spp**.	***Salmonella* spp**.	***Shigella* spp**.	***Staphylococcus* spp**.	***Listeria* spp**.	**Yeasts/ molds**
**log CFU/ g**								
AFPE	0.54 ± 0.18	(-)	0.34 ± 0.02	(-)	(-)	0.51 ± 0.19	0.11 ± 0.03	(-)/(-)
AFPU	0.55 ± 0.16	(-)	(-)	(-)	(-)	0.12 ± 0.33	(-)	(-)/(-)
AMPE	3.62 ± 0.18	2.93 ± 0.17	2.62 ± 0.02	1.93 ± 0.37	2.53 ± 0.15	0.89 ± 0.34	3.64 ± 0.19	(-)/ 2.25 ± 0.22
AMPU	3.68 ± 0.37	(-)	2.62 ± 0.03	0.55 ± 0.24	1.00 ± 0.24	1.52 ± 0.35	0.55 ± 0.024	(-)/ 2.35 ± 0.37
CPE	3.85 ± 0.38	(-)	(-)	(-)	(-)	3.68 ± 0.36	4.58 ± 0.05	(-)/ 4.53 ± 0.38

Data represent mean ± standard deviation of three experimental repetitions. (-): not detected. AFPE, immature firm light green peel (ready to eat in 4 days); AFPU, pulp from immature firm light green fruits (ready to eat in 4 days); AMPE, peel from mature intense green (ready to eat) fruit; AMPU: pulp from mature intense green (ready to eat) fruit; CPE, bulk of peel and pulp from visibly spoiled fruits.

### Antibiotic resistance pattern

**Figure 5:**
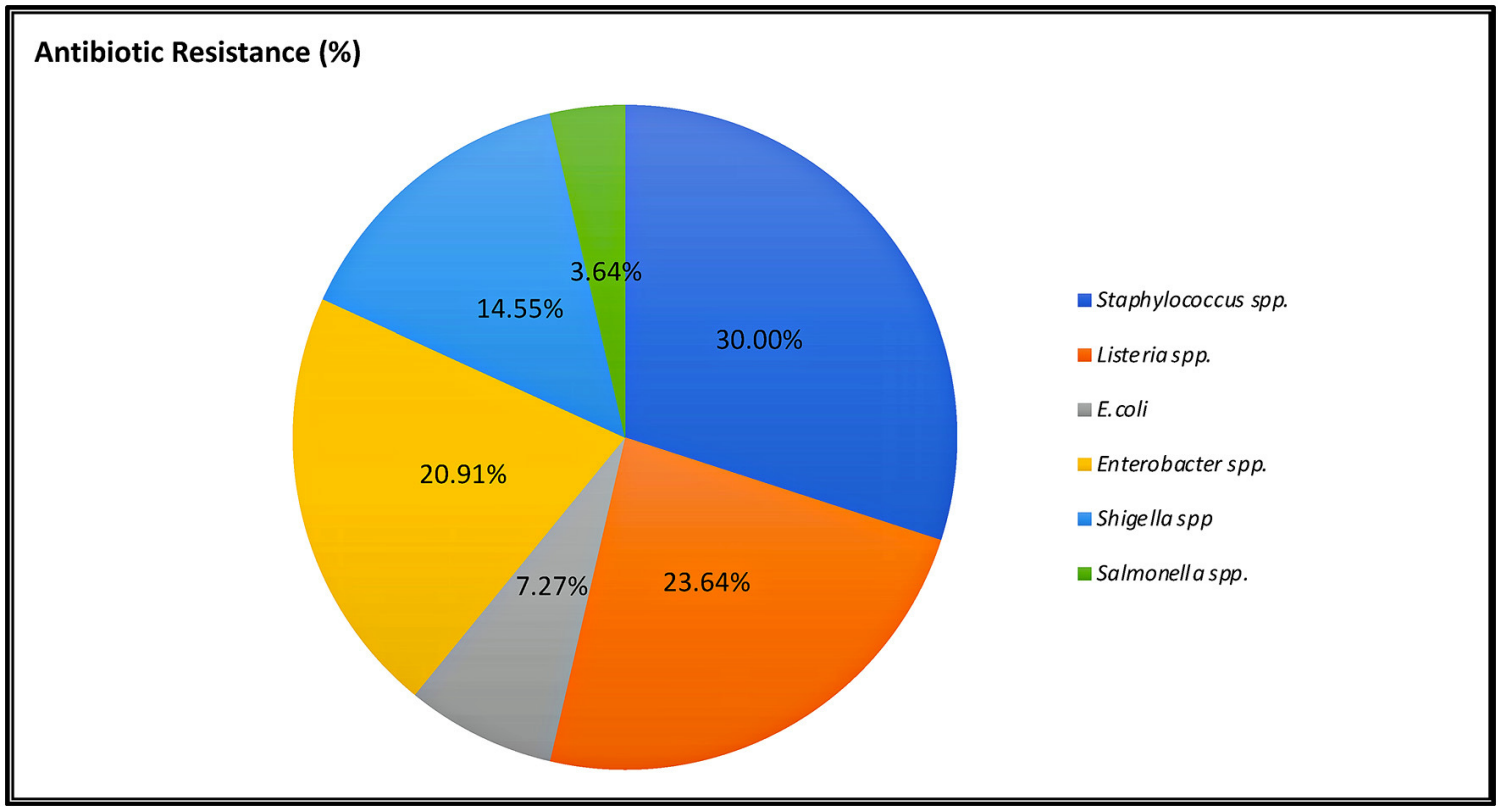
Percentage of indicator selected clones showing resistance to at least three classes of antibiotics.

**Figure 6:**
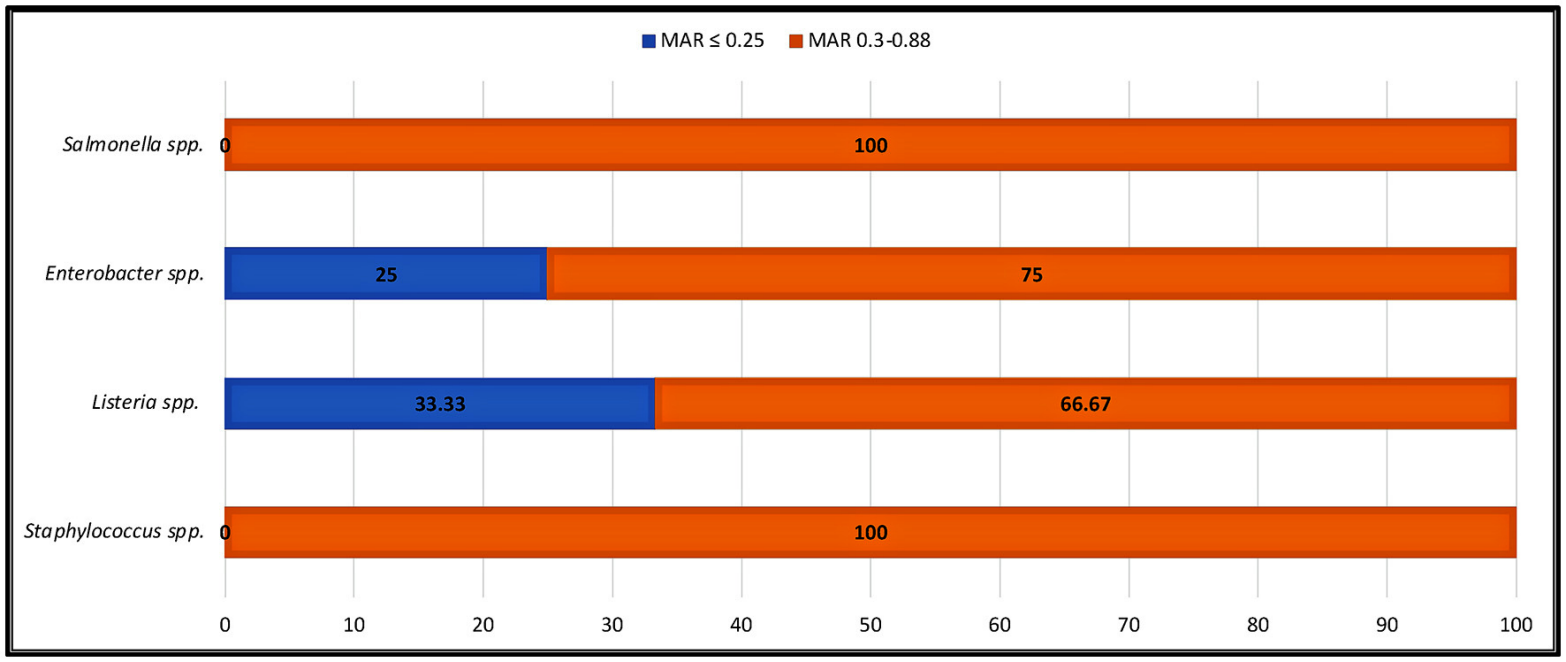
Percentage of indicator strains showing high MAR index. MAR index is calculated as the ratio between the number of antibiotics that an isolate is resistant to and the total number of antibiotics the organism is exposed to.

Antibiotic resistance is a global issue (Ayandele et al., 2020). Currently, there is substantial proof that the improper management of contamination has resulted in a severe problem with MAR (Catalano et al., 2022). The percentage of AR resistance among STAPHY, LIST, ENT, and SALM clones isolated from avocado fruits is shown in Figure 5. In addition, the prevalence of AR among isolates from various classes of antibiotics, such as tetracycline (TE30), aminoglycosides (K30, CN10), cephalosporins (VAN30), beta-lactamases (AM10), and penicillin-like antibiotics (AX25, MET5), is shown in Supplementary Tables 4–7. This research revealed that out of 21 selected STAPHY clones, only one was methicillin sensitive but resistant to four different antibiotic classes (Supplementary Table 4). Additionally, 13 clones were resistant to vancomycin. Similarly, among LIST, 11 clones were resistant to vancomycin and 6 to tetracycline (Supplementary Table 5). Among ENT clones, 9 were resistant to kanamycin (Supplementary Table 6). Although only 4 SALM clones were detected in mature fruits (AMPE and AMPU), they were resistant to amoxicillin (Supplementary Table 7). Moreover, the MAR index was calculated for each clone (Supplementary Tables 4–7). The MAR index is an efficient, reliable, and effective method for locating the sources of antibiotic-resistant bacteria (Davis and Brown, 2016). According to previous studies, a MAR >0.2 denotes a source of contamination with significant risk (Davis and Brown, 2016). However, this study found that 100% of STAPHY clones, 66.67% of LIST clones, and 75% of ENT clones had MAR indices >0.25 (Figure 6). In addition, complementary hemolysis and gelatinase assays indicated that 100% of the selected STAPHY clones showed beta-hemolysis and were positive for the presence of enzyme gelatinase (data not shown). Previous research indicates that hemolysin and gelatinase from *Staphylococcus* are significant virulence factors with cytotoxic actions (Bertelloni et al., 2021).

### Taxonomy assignment of STAPHY and ENT clones

The 16S rRNA sequencing of the nine selected STAPHY clones showing a MAR index above 0.63 was identified as *S. xylosus* (clones A1 and A2), *S. saprophyticus* (clones A3, A4, and A8), *Mammaliicoccus sciuri* (clone A10), and *S. epidermis* (clones A5 and A11). Clone A6 presented no match in the GenBank database. *S. xylosus* is a ubiquitous species that lives on the mucous membranes and epithelium of animals, especially mammals (Nagase et al., 2002). Although this species is defined as non-pathogenic, a few strains were related to animal opportunistic infections (Siqueira and Lima, 2002; Dordet-Frisoni et al., 2007). BLASTN analysis against the 16S ribosomal RNA NCBI database indicated a 98.83% sequence identity of the A1 clone with *S. caeli* strain 82B (NR_180106.1) isolated from air sampling of an industrial rabbit held in Italy (MacFadyen et al., 2019). A2, A3, A4, and A8 clones showed 99.42% to 99.85% sequence identity with *S. saprophyticus* subsp. *saprophyticus* ATCC 15305 (NR_074999.2) isolated from urine, *S. edaphicus* strain CCM8730 (NR_156818.1) isolated in Antarctica (Pantuček et al., 2018), and *S. casei* strain SB72 (NR_037053.1) isolated from ripened cheese (Place et al., 2002). In addition, *S. saprophyticus* was found as a food contaminant with a high prevalence of 34% in beef and pork meat (Hedman et al., 1990). Clone A10, found in mature pulp, showed 100% sequence identity with *M. sciuri* strain DSM 20345 (NR_025520.1), a mesophilic human pathogen that was isolated from the skin of eastern gray squirrels (Jayne et al., 2015). Former known as *S. sciuri, M. sciuri* belongs to the novel order Mammaliicoccus and is a commensal animal-associated bacterium but is also found in food (Marino et al., 2011; Madhaiyan et al., 2020). These strains showed 97% sequence similarity with *M. sciuri* strain B9-58B isolated from retail pork meat products (Neyaz et al., 2020). Interestingly, clone A5 showed 100% sequence identity with *S. epidermis* clone D02 (GU003840.1) isolated from active sludge (Parsley et al., 2010), whereas clone A11 showed 98.11% identity with *S. epidermis* clone 2322 (MT604781.1). Additionally, two ENT clones (AMPE clone 14 and AMPU clone 15) showing the highest MAR index (0.83) were identified as *Enterobacter* sp. with 96.44% and 99.37% sequence identity with *Enterobacter bacterium* strain 35 (KY681875.1) found in forest mushrooms (Pent et al., 2017). We stipulate that the presence of these microorganisms may be related to storage facilities as the fruits are deposited directly on soil or wet cellars where small animals can cross through, as well as human manipulation, but this statement needs to be supported by further analysis. These isolates were deposited at the NCBI Gene Bank database with the following accession numbers: OQ372998 (*S. xylosus* FMCShyA1, https://www.ncbi.nlm.nih.gov/nuccore/2439391154), OQ372999 (*S. xylosus* FFCShyA2, https://www.ncbi.nlm.nih.gov/search/all/?term=OQ372999), OQ373000 (*S. saprophyticus* FFCShyA3, https://www.ncbi.nlm.nih.gov/search/all/?term=OQ373000.1), OQ373001 (*S. saprophyticus* FFCShyA4, https://www.ncbi.nlm.nih.gov/search/all/?term=OQ373001), OQ876755 (*S. epidermis* dFMCShy5, https://www.ncbi.nlm.nih.gov/search/all/?term=OQ876755), OQ373002 (*S. saprophyticus* FMCShyA8, https://www.ncbi.nlm.nih.gov/search/all/?term=OQ373002), OQ373003 (*M. sciuri* FMCShyA10, https://www.ncbi.nlm.nih.gov/search/all/?term=OQ373003), OQ876756 (*S. epidermis* FMCShy11, https://www.ncbi.nlm.nih.gov/search/all/?term=OQ876756), OQ876757 (*Enterobacter* sp. dFMCEag14, https://www.ncbi.nlm.nih.gov/search/all/?term=OQ876757), and OQ876758 (*Enterobacter sp*. dFMCEag15, https://www.ncbi.nlm.nih.gov/search/all/?term=OQ876758).

## Conclusion

This is the first study evaluating the microbiota and its antibiotic resistance profile in immature (firm) and mature (ready to eat) avocado Guatemalan fruits sold at retail markets in Ecuador. Although there were differences at the species level, 16S rRNA gene metagenomic data agree with the cultivable analyses at the genus level. Among several identified indicator microorganisms, some *Staphylococcus* sp. and *Enterobacter* sp. clones displayed resistance to various antibiotics. This preliminary study shows a core community of both useful and harmful bacteria in avocado fruits and offers crucial baseline information for further investigation of bacterial population variation transition from firm to mature stage. It is essential to ensure appropriate handling practices from the producer (farm) to the retail market; otherwise, a significant number of pathogens from the fruit surface may survive and spread to the pulp, posing a risk to consumer health. Thus, to achieve an increase in the production and marketing of the Fuerte cultivar in Ecuador, it is important to consider valuable strategies to protect the fruits at the early ripe stage in future. The current study might help the national authorities to reconsider the legislation on fresh fruits and vegetables handling and storage at the retail markets and establish effective control measures to prevent the spread of hazardous bacteria.

## Data availability statement

The original contributions presented in the study are included in the article/Supplementary material, further inquiries can be directed to the corresponding author.

## Author contributions

GT: conceptualization, methodology, writing—original draft preparation, project administration, and funding acquisition. GT and EA: investigation. GT and PC: software and reviewing and editing. All authors contributed to the article and approved the submitted version.
