# Supplementary material for: Microbiota and its antibiotic resistance profile in avocado Guatemalan fruits (Persea nubigena var. guatemalensis) sold at retail markets of Ibarra city, northern Ecuador
Source: Front Microbiol. 2023 Sep 7;14:1228079. doi: 10.3389/fmicb.2023.1228079 (PMC10513466; doi:10.3389/fmicb.2023.1228079)
Supplement: Supplementary file 1 [file Data_Sheet_1.docx]

Supplementary Material

Microbiota and its antibiotic resistance profile in avocado Guatemalan fruits (*Persea nubigena*var.*guatemalensis*) sold at retail markets of Ibarra city, Northern Ecuador

Evelyn Angamarca^1^, Pablo Castillejo^1,2^, and Gabriela N. Tenea^1^*

^1^Biofood and Nutraceutics Research and Development Group. Faculty of Engineering in Agricultural and Environmental Sciences, Universidad Técnica del Norte, 100150 Ibarra, Ecuador.

^2^ Grupo de Investigación en Biodiversidad, Medio Ambiente y Salud. Universidad de Las Américas, 170125 Quito, Ecuador.

*** Correspondence:**Corresponding Author: Gabriela N. Tenea
gntenea@utn.edu.ec

# Supplementary Tables

**Supplementary Table 1.** Top 10 bacterial communities at Phylum level

|  | AFPE | | AFPU | | AMPE | | AMPU | | CPE | |
| --- | --- | --- | --- | --- | --- | --- | --- | --- | --- | --- |
| Classification | **Number of Reads** | **% Total Reads** | **Number of Reads** | **% Total Reads** | **Number of Reads** | **% Total Reads** | **Number of Reads** | **% Total Reads** | **Number of Reads** | **% Total Reads** |
| Cyanobacteria/Chloroplast | 30,271 | 53.84 | 38,639 | 66.49 | 36,960 | 69.60 | 33,467 | 73.98 | 42,777 | 70.95 |
| Acidobacteria | 12,032 | 21.40 | 14,887 | 25.62 | 11765 | 22.15 | 8,222 | 18.18 | 12,887 | 21.37 |
| Proteobacteria | 7,183 | 12.77 | 2,186 | 3.76 | 1876 | 3.53 | 1,650 | 3.65 | 2,162 | 3.59 |
| Actinobacteria | 2,059 | 3.66 | 221 | 0.38 | 274 | 0.52 | 207 | 0.46 | 321 | 0.53 |
| Unclassified at Phylum level | 1,418 | 2.52 | 1,422 | 2.45 | 1383 | 2.60 | 1,083 | 2.39 | 1,394 | 2.31 |
| Firmicutes | 1,273 | 2.26 | 390 | 0.67 | 429 | 0.81 | 288 | 0.64 | 352 | 0.58 |
| Bacteroidetes | 976 | 1.74 | 129 | 0.22 | 93 | 0.18 | 70 | 0.15 | 114 | 0.19 |
| Planctomycetes | 260 | 0.46 |  |  |  |  |  |  |  |  |
| Armatimonadetes |  |  | 102 | 0.18 | 130 | 0.84 | 80 | 0.18 | 116 | 0.19 |
| Planctomycetes | 17 |  | 27 |  | 260 | 0.24 | 49 |  | 27 |  |

Legend: AFPE: immature firm light-green peel (ready to eat in 4 days); AFPU: pulp from immature firm light-green fruits (ready to eat in 4 days); AMPE: peel from mature intense green (ready to eat) fruit; AMPU: pulp from mature intense green (ready to eat) fruit; CPE: bulk of peel and pulp from visibly spoiled fruits.

**Supplementary Table 2.** Most abundant categories (%) at Genus level

| Genus category | AFPE | AFPU | AMPE | AMPU | CPE |
| --- | --- | --- | --- | --- | --- |
| Streptophyta | 53.29% | 66.06% | 69.13% | 73.34% | 70.38% |
| Gp15 | 20.62% | 25.39% | 21.96% | 17.87% | 21.13% |
| Unclassified | 5.19% | 4.17% | 4.36% | 4.27% | 3.92% |
| Plesiocystis | 1.07% | 1.19% | 0.56% | 1.11% | 1.00% |
| Streptomyces | 1.04% |  |  |  |  |
| Rhizobium | 0.94% |  |  |  |  |
| Sphingobium | 0.67% |  |  |  |  |
| Sphingomonas | 0.66% |  |  |  |  |
| Pleomorphobacterium |  | 0.60% | 0.37% | 0.60% | 0.54% |
| Chthonomonas/Armatimonadetes_gp3 |  | 0.16% | 0.21% | 0.15% | 0.16% |
| Acinetobacter |  | 0.14% |  |  |  |
| Limimonas |  | 0.09% |  |  |  |
| Escherichia/Shigella |  |  | 0.80% |  |  |
| Sporolactobacillus |  |  | 0.16% |  | 0.09% |
| Chlorophyta |  |  |  | 0.11% |  |
| GpXIII |  |  |  | 0.09% |  |
| Pantoea |  |  |  |  | 0.13% |
| Other | 16.52% | 2.20% | 2.45% | 2.46% | 2.65% |

Legend: AFPE: immature firm light green peel (ready to eat in 4 days); AFPU: pulp from immature firm light green fruits (ready to eat in 4 days); AMPE: peel from mature intense green (ready to eat) fruit; AMPU: pulp from mature intense green (ready to eat) fruit; CPE: bulk of peel and pulp from visibly spoiled fruits.

**Supplementary Table 3.** Physicochemical parameters of avocados

| **Sample** | **Total solids (°Brix)** | **pH** | **Acidity (tartaric acid %)** | **Maturity index (MI)** |
| --- | --- | --- | --- | --- |
| AF | 0.67 ± 0.05 | 6.36 ± 0.02 | 0.028 ± 0.15 | 22.81 ± 0.05 |
| AM | 1.91 ± 0.05 | 6.50 ± 0.05 | 0.041 ± 0.01 | 48.33 ± 0.24 |
| CPE | 2.41 ± 0.05 | 6.94 ± 0.05 | 0.051 ± 0.01 | 50.70 ± 0.24 |

Data are means ± standard error. Legend: AF: immature fruit; AM: mature fruit; CPE: bulk of peel and pulp from visible spoiled fruits.

**Supplementary Table 4**. Antibiotic susceptibility pattern and MAR index of *Staphylococcus* spp. selected clones

| **Samples** | | **No. of antibiotics that the clone was resistant** | **Diameter of the inhibition zone (mm)** | | | | | | | | | **MAR** |
| --- | --- | --- | --- | --- | --- | --- | --- | --- | --- | --- | --- | --- |
|  |  |  | **Tetracycline** | | **Cephalosporins** | | **b-lactam-Lactamase inhibitor combinations** | **Aminoglycosides** | | **Penicillin like antibiotics** | |  |
|  |  |  | **TE30** | | **VAN30** | **CXM30** | **AM10** | **K30** | **CN10** | **AX25** | **MET5** |  |
| AMPE clone A1 | | 7 | 23.40 | | 8.60 | 9.40 | 10.40 | 8.70 | 9.10 | 10.40 | 13.50 | 0.88 |
| AMPE clone A3 | | 7 | 19.00 | | 10.00 | 6.00 | 6.00 | 12.00 | 9.00 | 11.00 | 6.00 | 0.88 |
| AFPE clone A2 | | 7 | 18.00 | | 13.70 | 6.00 | 8.00 | 8.60 | 9.80 | 6.00 | 7.00 | 0.88 |
| AFPE clone A4 | | 6 | 19.00 | | 6.00 | 18.00 | 6.00 | 12.00 | 7.00 | 6.00 | 6.00 | 0.75 |
| AMPU clone A8 | | 6 | 20.50 | | 13.60 | 10.10 | 8.70 | 11.10 | 12.80 | 6.00 | 6.00 | 0.75 |
| AMPE clone A5 | | 5 | 17.00 | | 11.00 | 13.00 | 8.00 | 19.00 | 13.00 | 11.00 | 6.00 | 0.63 |
| AMPE clone A6 | | 5 | 20.50 | | 10.00 | 18.60 | 9.20 | 12.00 | 12.10 | 10.10 | 23.90 | 0.63 |
| AMPE clone A11 | | 5 | 6.00 | | 8.00 | 20.00 | 14.00 | 17.00 | 10.00 | 6.00 | 6.00 | 0.63 |
| AMPU clone A10 | | 5 | 13.00 | | 12.00 | 13.00 | 17.00 | 12.00 | 18.00 | 18.00 | 6.00 | 0.63 |
| AMPE clone A17 | | 4 | 29.30 | | 14.30 | 31.50 | 12.80 | 25.70 | 27.60 | 9.80 | 6.00 | 0.50 |
| AMPE clone A8 | | 4 | 26.00 | | 7.00 | 25.00 | 6.00 | 22.00 | 10.00 | 22.00 | 12.00 | 0.50 |
| AMPU clone A14 | | 4 | 36.40 | | 12.70 | 18.60 | 14.40 | 27.60 | 12.10 | 8.60 | 10.60 | 0.50 |
| AMPU clone A16 | | 4 | 20.60 | | 15.70 | 13.90 | 11.90 | 14.60 | 13.80 | 6.00 | 12.90 | 0.50 |
| AMPU clone A7 | | 4 | 15.00 | | 8.00 | 25.00 | 12.00 | 15.00 | 9.00 | 16.00 | 7.00 | 0.50 |
| AFPU clone A18 | | 4 | 19.00 | | 6.00 | 18.00 | 7.00 | 14.00 | 14.00 | 11.00 | 6.00 | 0.50 |
| AMPE clone A13 | | 3 | 26.00 | | 10.80 | 18.00 | 9.00 | 30.00 | 13.20 | 16.00 | 14.70 | 0.38 |
| AFPE clone A20 | | 3 | 10.00 | | 9.00 | 16.00 | 17.00 | 17.00 | 17.00 | 19.00 | 7.00 | 0.38 |
| AMPE clone A12 | | 2 | 23.00 | | 12.30 | 27.00 | 16.00 | 20.00 | 13.00 | 21.00 | 12.90 | 0.25 |
| AFPE clone A19 | | 2 | 22.00 | | 10.00 | 24.00 | 26.00 | 17.00 | 16.00 | 17.00 | 16.00 | 0.25 |
| AFPE clone A22 | | 2 | 35.00 | | 21.00 | 35.00 | 35.00 | 22.00 | 27.00 | 38.00 | 7.00 | 0.25 |
| AFPU clone A21 | | 2 | 7.00 | | 15.00 | 26.00 | 23.00 | 23.00 | 24.00 | 25.00 | 7.00 | 0.25 |
| *Staphylococcus aureus* ATCC 1026 | | 7 | 26.00 | | 13.00 | 6.00 | 6.00 | 6.00 | 6.00 | 6.00 | 6.00 | 0.88 |
| *Staphylococcus aureus* ATCC 43300 | | 6 | 26.00 | | 12.80 | 18.00 | 6.00 | 6.00 | 6.90 | 7.00 | 17.70 | 0.75 |
|  | Resistant | | |  |  |  |  |  |  |  |  |  |
|  | Intermediate resistant | | |  |  |  |  |  |  |  |  |  |
|  | Sensible | | |  |  |  |  |  |  |  |  |  |

Legend: AFPE: immature firm light green peel (ready to eat in 4 days); AFPU: pulp from immature firm light green fruits (ready to eat in 4 days); AMPE: peel from mature intense green (ready to eat) fruit; AMPU: pulp from mature intense green (ready to eat) fruit.

**Supplementary Table 5.** Antibiotic susceptibility pattern and MAR index of *Listeria* spp. selected clones

| **Samples** | **No. of antibiotics that the clone was resistant** | | **Diameter of the inhibition zone (mm)** | | | | | | | | **MAR** |
| --- | --- | --- | --- | --- | --- | --- | --- | --- | --- | --- | --- |
|  |  |  | **Tetracycline** | | **Cephalosporins** | | **b-lactam-Lactamase inhibitor combinations** | **Aminoglycosides** | | **Penicillin like antibiotics** |  |
|  |  |  | **TE30** | | **VAN30** | **CXM30** | **AM10** | **K30** | **CN10** | **AX25** |  |
| AMPE clone 3 | 4 | | 8.00 | | 11.00 | 28.00 | 13.00 | 18.00 | 20.00 | 15.00 | 0.57 |
| AMPU clone 1 | 4 | | 20.20 | | 10.80 | 19.00 | 8.80 | 14.20 | 6.00 | 10.20 | 0.57 |
| AMPU clone 1 | 4 | | 7.00 | | 11.00 | 25.00 | 17.00 | 18.00 | 19.00 | 17.00 | 0.57 |
| AMPU clone 3 | 3 | | 8.70 | | 6.00 | 23.00 | 19.00 | 11.80 | 14.00 | 25.00 | 0.43 |
| AMPE clone 5 | 3 | | 20.70 | | 15.10 | 19.00 | 9.10 | 14.20 | 6.00 | 10.20 | 0.43 |
| AMPU clone 7 | 3 | | 24.00 | | 13.00 | 23.00 | 13.00 | 20.00 | 18.00 | 16.00 | 0.43 |
| AMPU clone 12 | 3 | | 8.70 | | 13.60 | 22.80 | 22.60 | 11.60 | 13.80 | 25.30 | 0.43 |
| AMPU clone 2 | 2 | | 26.00 | | 13.00 | 27.00 | 17.00 | 7.00 | 15.00 | 21.00 | 0.29 |
| AMPU clone 9 | 2 | | 29.00 | | 13.30 | 29.80 | 17.00 | 19.30 | 20.00 | 11.30 | 0.29 |
| AMPU clone 11 | 2 | | 30.00 | | 13.00 | 29.00 | 13.00 | 21.00 | 27.00 | 14.00 | 0.29 |
| AMPE clone 4 | 1 | | 24.00 | | 12.00 | 27.00 | 16.00 | 19.00 | 15.00 | 20.00 | 0.14 |
| AMPE clone 6 | 1 | | 26.00 | | 15.00 | 23.00 | 15.00 | 18.00 | 15.00 | 26.00 | 0.14 |
| AMPU clone 8 | 1 | | 24.00 | | 18.00 | 25.00 | 24.00 | 18.00 | 14.00 | 18.00 | 0.14 |
| AMPU clone 10 | 1 | | 25.80 | | 11.70 | 21.20 | 20.10 | 15.80 | 15.00 | 18.90 | 0.14 |
| AMPU clone 12 | 0 | | 40.00 | | 25.00 | 40.00 | 41.00 | 16.00 | 27.00 | 45.00 | 0.00 |
| *Listeria monocytogenes* ATCC 19115 | 3 | | 7.00 | | 20.00 | 7.00 | 11.00 | 18.00 | 17.10 | 26.00 | 0.43 |
|  | | Resistant | |  |  |  |  |  |  |  |  |
|  | | Intermediate resistant | |  |  |  |  |  |  |  |  |
|  | | Sensible | |  |  |  |  |  |  |  |  |

Legend: AFPE: immature firm light-green peel (ready to eat in 4 days); AFPU: pulp from immature firm light green fruits (ready to eat in 4 days); AMPE: peel from mature intense green (ready to eat) fruit; AMPU: pulp from mature intense green (ready to eat) fruit.

**Supplementary Table 6.** Antibiotic susceptibility pattern and MAR index of *Enterobacter* spp. selected clones

| **Samples** | | **No. of antibiotics that the clone was resistant** | **Diameter of the inhibition zone (mm)** | | | | | | | **MAR** |
| --- | --- | --- | --- | --- | --- | --- | --- | --- | --- | --- |
|  |  |  | **Tetracycline** | | **Cephalosporins** | **b-lactam-Lactamase inhibitor combinations** | **Penicillin like antibiotics** | **Aminoglycosides** | |  |
|  |  |  | **TE30** | | **CXM30** | **AM10** | **AX25** | **K30** | **CN10** |  |
| AMPE clone 14 | | 5 | 20.80 | | 11.00 | 9.30 | 8.70 | 8.60 | 11.20 | 0.83 |
| AMPU clone 15 | | 5 | 14.30 | | 9.80 | 8.60 | 6.00 | 8.60 | 11.70 | 0.83 |
| AMPE clone 7 | | 3 | 23.00 | | 19.00 | 12.00 | 18.00 | 13.00 | 9.00 | 0.50 |
| AMPU clone 10 | | 3 | 9.00 | | 20.00 | 12.00 | 16.00 | 12.00 | 15.00 | 0.50 |
| AMPU clone 11 | | 3 | 10.00 | | 22.00 | 15.00 | 15.00 | 12.00 | 14.00 | 0.50 |
| AMPU clone 12 | | 3 | 19.00 | | 21.00 | 9.00 | 10.00 | 12.00 | 13.00 | 0.50 |
| AMPE clone 13 | | 3 | 19.10 | | 10.80 | 18.00 | 9.20 | 10.20 | 15.00 | 0.50 |
| AMPE clone 5 | | 2 | 20.00 | | 16.00 | 6.00 | 16.00 | 14.00 | 6.00 | 0.33 |
| AMPE clone 9 | | 2 | 22.00 | | 17.00 | 12.00 | 19.00 | 13.00 | 18.00 | 0.33 |
| AMPE clone 6 | | 1 | 24.00 | | 18.00 | 15.00 | 6.00 | 15.00 | 18.00 | 0.17 |
| AMPU clone 8 | | 1 | 22.00 | | 22.00 | 16.00 | 18.00 | 13.00 | 16.00 | 0.17 |
| AFPE clone 17 | | 1 | 23.00 | | 19.00 | 12.00 | 15.00 | 17.00 | 17.00 | 0.17 |
| *Enterobacter spp. (laboratory isolate from strawberry fruit)* | | 5 | 7.00 | | 12.00 | 12.00 | 15.00 | 10.00 | 10.00 | 0.83 |
| *E.coli* ATCC 25922 | | 2 | 20.00 | | 23.00 | 15.00 | 9.00 | 12.00 | 20.00 | 0.33 |
|  | Resistant | | |  |  |  |  |  |  |  |
|  | Intermediate resistant | | |  |  |  |  |  |  |  |
|  | Sensible | | |  |  |  |  |  |  |  |

Legend: AFPE: immature firm light green peel (ready to eat in 4 days); AFPU: pulp from immature firm light green fruits (ready to eat in 4 days); AMPE: peel from mature intense green (ready to eat) fruit; AMPU: pulp from mature intense green (ready to eat) fruit.

**Supplementary Table 7.** Antibiotic susceptibility pattern and MAR index of *Salmonella* spp. selected clones.

| **Description** | **No. of antibiotics that the clone was resistant** | | **Diameter of the inhibition zone (mm)** | | | | | | | **MAR** |
| --- | --- | --- | --- | --- | --- | --- | --- | --- | --- | --- |
|  |  |  | **Tetracycline** | | **Cephalosporins** | **b-lactam-Lactamase inhibitor combinations** | **Penicillin like antibiotics** | **Aminoglycosides** | |  |
|  |  |  | **TE30** | | **CXM30** | **AM10** | **AX25** | **K30** | **CN10** |  |
| AMPE clone 1 | 4 | | 28.00 | | 18.00 | 6.00 | 6.00 | 12.00 | 9.00 | 0.67 |
| AMPU clone 4 | 4 | | 24.00 | | 12.00 | 15.00 | 8.00 | 6.00 | 17.00 | 0.67 |
| AMPE clone 2 | 2 | | 21.60 | | 27.60 | 22.70 | 14.60 | 21.60 | 12.10 | 0.33 |
| AMPU clone 3 | 3 | | 23.00 | | 18.00 | 12.00 | 6.00 | 10.00 | 13.00 | 0.50 |
| *Salmonella enterica* subsp. *enterica* ATCC 51741 | 5 | | 16.00 | | 6.00 | 15.00 | 12.00 | 8.00 | 16.00 | 0.83 |
|  | | Resistant | |  |  |  |  |  |  |  |
|  | | Intermediate resistant | |  |  |  |  |  |  |  |
|  | | Sensible | |  |  |  |  |  |  |  |

MAR index was calculated as the ratio between the number of antibiotics that an isolate is resistant to and the total number of antibiotics the organism is exposed to. Legend: AFPE: immature firm light green peel (ready to eat in 4 days); AFPU: pulp from immature firm light green fruits (ready to eat in 4 days); AMPE: peel from mature intense green (ready to eat) fruit; AMPU: pulp from mature intense green (ready to eat) fruit.

## Supplementary Figures

A). B).


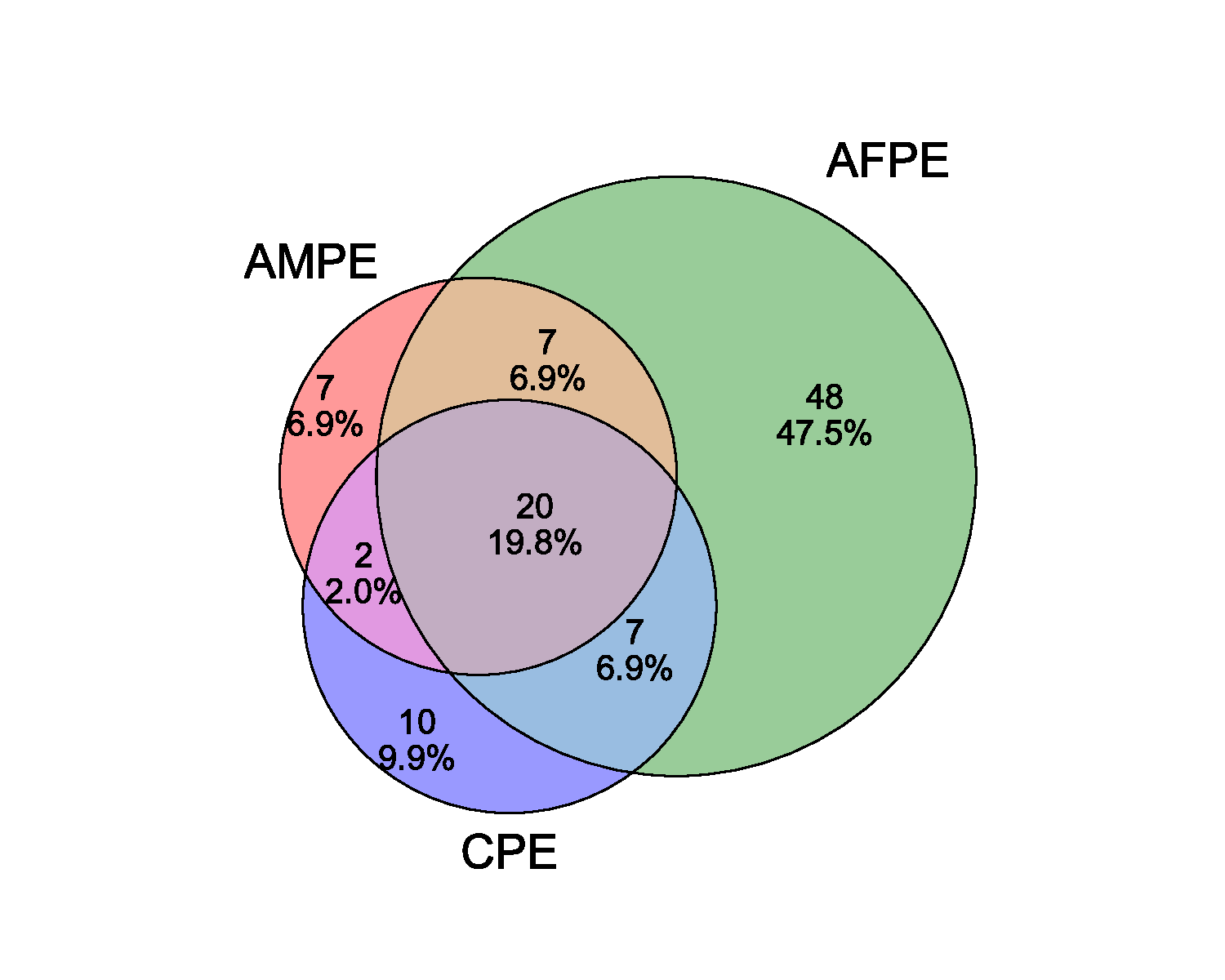

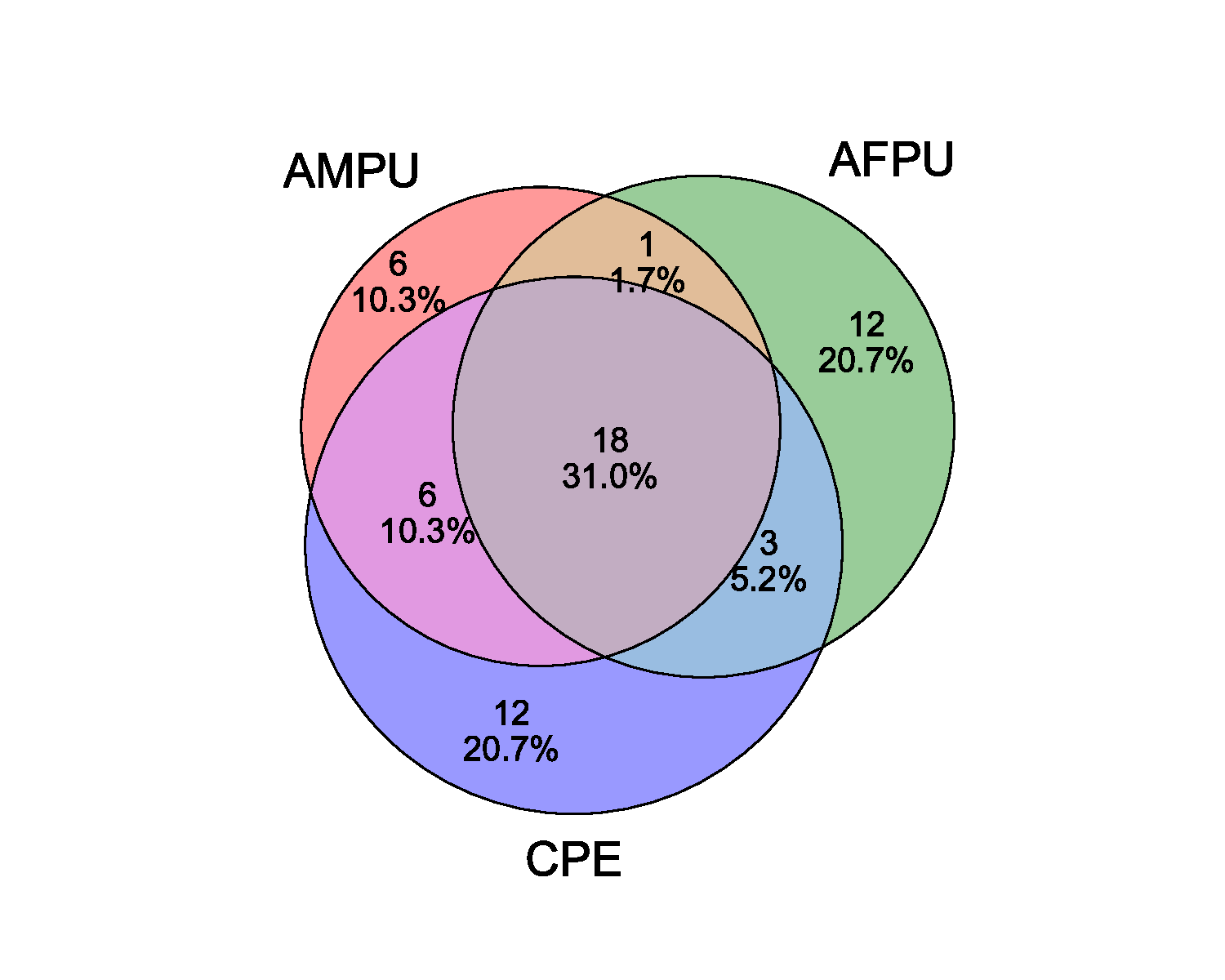


**Supplementary Figure 1.** Venn diagram showing the number and percentage of shared bacterial families among A) peel; B) pulp. Legend: AFPE: immature firm light green peel (ready to eat in 4 days); AFPU: pulp from immature firm light green fruits (ready to eat in 4 days); AMPE: peel from mature intense green (ready to eat) fruit; AMPU: pulp from mature intense green (ready to eat) fruit; CPE: bulk of peel and pulp from visibly spoiled fruits.

**
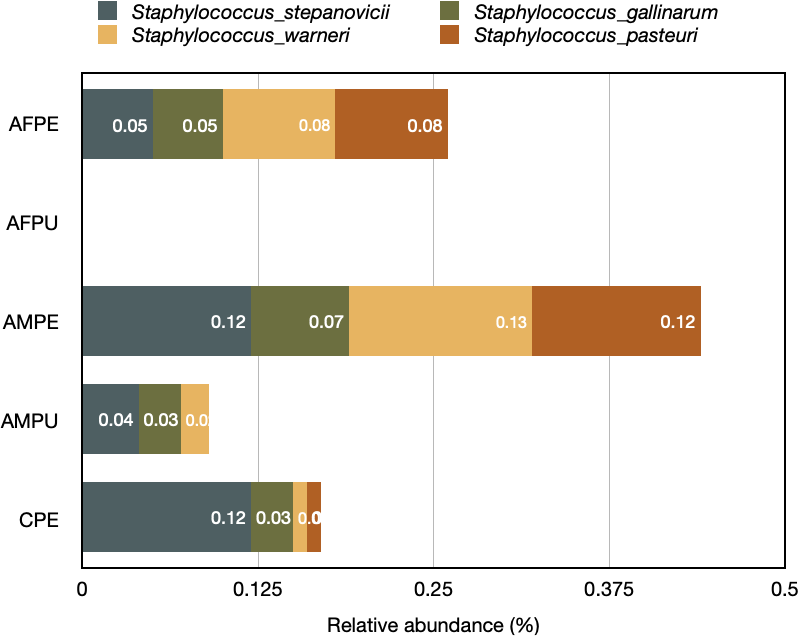
**

**Supplementary Figure 2.** Relative abundance (%) of *Staphylococcus* species detected among the groups. AFPE: immature firm light green peel (ready to eat in 4 days); AFPU: pulp from immature firm light green fruits (ready to eat in 4 days); AMPE: peel from mature intense green (ready to eat) fruit; AMPU: pulp from mature intense green (ready to eat) fruit; CPE: bulk of peel and pulp from visibly spoiled fruits.
